# The use of various appointment systems among patients visiting academic outpatient centers in Kerman and the evaluation of patients’ perspective and satisfaction

**DOI:** 10.1186/s12913-022-08635-6

**Published:** 2022-11-14

**Authors:** Fatemeh Bagheri, Farzaneh Behnam, Zahra Galavi, Leila Ahmadian

**Affiliations:** Department of Health Information Sciences, Faculty of Management and Medical Information Sciences, Kerman University of Medical Sciences, Kerman, Iran

**Keywords:** Appointment systems, Patient appointment, Patient schedules, Evaluation, Satisfaction

## Abstract

**Background:**

To optimize appointment systems, it is necessary to assess their users’ perspectives. This study aims to determine the use of various appointment systems among patients in academic outpatient centers and to investigate their perspectives and satisfaction.

**Methods:**

This survey study was conducted on 332 patients or those accompanying patients in academic outpatient centers. A five-part questionnaire consisting of (1) demographic information, (2) willingness to use systems, (3) problems when using these systems, (4) problems after reserving the appointment, (5) recommendations and critics was used. The relationship between the system of interest and the available tools was examined by the Chi-square test, and the relationship between demographic characteristics and satisfaction was assessed using multiple regression.

**Results:**

The participants’ overall satisfaction towards appointment systems, regardless of the type of system, was 49.12 ± 16.04 (out of 100). Satisfaction with the appointment system using Unstructured Supplementary Service Data (USSD) was significantly higher than the other two systems (p = 0.03). Web-based application and Interactive Voice Response (IVR) were the most frequently used systems with 61% and 48%, respectively. More than half of those who had access to a telephone (56%) preferred the IVR appointment system, and most of those who had Internet access (71%) preferred the web-based application (p < 0.05). Among 137 participants who had access to both the Internet and telephone, 49% (n = 67) stated that they would rather arrange their appointment through the web-based application.

**Conclusion:**

The web-based application and IVR are the most frequently used and favorable appointment system among the patients or those accompanying patients. Despite the availability of the infrastructure, the participant had moderate satisfaction with these systems due to their failures. Therefore, to have more efficient systems and increase patients or those accompanying patients satisfaction with these systems, healthcare authorities should have a plan to solve the problems of these systemes and use the capacity of information resources to inform the community regarding these systems.

## Background

To receive health services, patients have the most interaction with outpatient services. Patients’ satisfaction with outpatient services is influenced by factors such as quality of health services, ease of appointment, and access to required services [1–4]. Therefore, one of the issues for providing services to patients in outpatient care is planning and scheduling services for patients with the least waste of time [5]. In the past, the process of scheduling outpatient centers has been done by walk-in patients [1]. One of the biggest challenges of this traditional manner is the long waiting time in the queue to receive services [5]. A study shows that a non-user friendly appointment system is the main barrier to patients’ medical follow-up compliance [6].

With the advent of information technology and its growing use, especially in the field of health, many outpatient centers use appointment systems. These systems are designed to provide better services and speed up the workflow. An efficient appointment system can reduce waiting time, increase patient satisfaction, and save resources [2, 7–11]. Today, various systems are available for patients or those accompanying patients to schedule their appointments in health care centers. Interactive Voice Response (IVR) is one of these systems that provide information to users through voice messages and receive appropriate responses from users, allowing their subscribers to schedule their appointment [12]. The Internet-based appointment systems allow the individuals to have a contactless booking through a communication network [7]. The two major types of internet-based appointment services include scheduling software as a service and proprietary Web-based scheduling systems [1]. Unstructured Supplementary Service Data (USSD) is another approach, which like IVR, is accessible through the telephone. USSD provide a structured menu to users by entering a numerical code in the mobile phone and allowing their subscribers to schedule their appointment by entering appropriate answers [13].

Kerman is the largest province and the most populated city in the southeast, Iran [14]. Due to the advancement of medicine in this city, some healthcare centers in this city are referral centers for providing care in the region compared to other cities in this province and even neighboring provinces. On a daily basis, many patients are referred to the outpatient centers affiliated with Kerman University of Medical Sciences (KUMS). Therefore, the high volume of patients and arranging their appointments are major challenges in these centers as the face-to-face manner lead to long waiting time for walk-in patients. KUMS with the aim of facilitating the provision of healthcare services to patients or those accompanying patients and in order to regulate their appointment has launched three appointment systems; web-based application, USSD, and IVR. To better manage the work processes of the healthcare centers and upgrade the developed systems, it is necessary to assess the views of their users. Therefore, the purpose of this study was to determine the use of the appointment systems by patients visiting the outpatient centers and to examine the perspective and the satisfaction of the users of these systems. The results of this study can help health care authorities to have better appointment systems to meet the needs of the community and improve the work process.

## Methods

### Study design

This survey study is conducted in Kerman. Out of six outpatient centers affiliated to KUMS, two centers (providing specialized and sub-specialized services) were randomly selected as the sampling settings. The patients or those accompanying patients of these centers could schedule their appointment through either the appointment systems or walk-in.

The types of appointment systems available in these centers include Web-based application, IVR and USSD appointment system. A web-based application that allows individuals to conveniently and securely book their appointments and reservations online through any Web-connected device [15]. IVR, is an automated telephone system that combines pre-recorded messages or text-to-speech technology with a Dual Tone Multi Frequency (DTMF) interface to engage callers, allowing them to provide and access information without a live agent [16]. USSD, is a protocol used by Global System for Mobile (GSM) Communications cellular telephones to communicate with the service provider’s computers [17]. In USSD, users can access a structured menu by entering a numerical code in the mobile phone. They can navigate the options menu and book appointments using the mobile keyboard [13].

On average, these centers provide services to 35,000 patients per month. Patients attending their appointment were recruited. In case patient was unable to fill the questionnaire or did not make his appointment on his own, indivituals accompanying patients were recruited. The inclusion criteria were (1) ability to read and write, (2) patients or those accompany patients who used appointment systems and (3) Willingness to participate in the study. The sample size for this study was determined using Cochran’s formula of 380 people ( P = 0.5 ). One of the researchers went to the selected centers and randomly invited at least 30 patients or those accompanying patients to complete the questionnaire every time.

### Data collection tools

To collect data, three researchers-made questionnaires were developed. Each of these questionnaires consists the questions regarding one of the three appointment systems of the web-based application, USSD, and IVR separately. Six experts (two information technology specialists, two medical informatics specialists, and two health information specialists) confirmed the face and content validity of the questionnaires. The reliability of the questionnaires was also confirmed by calculating the internal correlation with Cronbach’s alpha (r = 0.87). The questionnaires consisted of five sections. The questions of all three questionnaires had the same structure and content in four sections, and only the questions of the third section were different among the questionnaires. The third section consists of the questions regarding the problems with the appointment system. Since each system has its specific functionality and workflow, the questions in this section were adjusted based on the type of appointment system. The sections of the questionnaire were as follows: (1) Demographic information including age, gender, level of education, and place of residence (4 questions); (2) Questions to measure the use and willingness of the participants to use the appointment system (5 questions); (3) Questions about the problems when using the system (this section includes 17 questions for the web-based application, 13 questions for IVR and USSD each) (4) Questions about the problems that participants faced after booking their appointment (5 questions) (5) There were two open-ended questions in the last section for providing the recommendations and critics. In total, 33 questions were used to assess the Web-based application and 29 questions for each the IVR and USSD systems.

The answer options were different based on the type of question. Thus, the answers to the questions in the first to fifth sections for the web-based application were as follows: eight questions with a five-point Likert scale from not at all to too many, five questions with yes and no options, seventeen multiple-choice questions, and three open-ended questions. Also, the answers to the questions in the first to fifth sections for each of the IVR and USSD systems were as follows: eight questions with a five-point Likert scale from not at all to too many, four questions with yes and no options, fourteen multiple-choice questions, and three open-ended questions.

The paper-based questionnaires were filled out in the researcher’s presence during the waiting period before the visit. If an invited patient or those accompanying patient did not want to complete the questionnaire, this person was replaced with a new patient or that accompanying patient.

### Data analysis

Data analysis was performed using SPSS 21 to analyze the data. The answers of the third and fourth sections of the questionnaire were scored between zero and 100 (not at all = 0, only once = 25, a few times = 50, many = 75, too many = 100). Two-choice questions were scored as follows: yes = 100 and no = 0. To estimate the overall satisfaction score of the participants, the average score of the questions in the third and fourth sections was used. To analyze the satisfaction scores, the level of satisfaction was considered in three categories: low (scores below 25), medium (scores between 25 and 75), and high (scores above 75). The Chi-square test was used to measure the relationship between the system of interest and available tools. The relationship between demographic characteristics and satisfaction was investigated by multiple regression. Moreover, to investigate the relationship between satisfaction and the type of appointment system the Analysis of Variance (ANOVA) test was used.

## Results

Out of 400 distributed questionnaires, 332 (83%) questionnaires were completed and used for analysis. Seventy-five% (n = 250) of the participants were in the age group of 20–40. About 45% (n = 150) of the participants had an associate’s degree or bachelor’s degree. Seventy-seven% (n = 256) of the participants live in Kerman (Table 1).

**Table 1:** Demographic information of the participants

Demographic information		n (%)
Age	< 20	24 (7.22)
	20–40	250 (75.30)
	41–60	53 (15.97)
	> 60	5 (1.51)
Gender	Female	243 (73.19)
	Male	89 (26.81)
Education	High-school diploma and under	146 (43.98)
	Associate’s degree and bachelor’s degree	150 (45.18)
	Master’s degree and higher	36 (10.84)
Place of residence	Kerman	256 (77.11)
	Cities of Kerman province	70 (21.08)
	Other (Tabriz, Bandar Abbas, Tehran, Zahedan)	6 (1.81)

The web-based application and IVR with 61% (n = 202) and 48% (n = 159) were the more frequently used appointment systems and the more favorable systems as well (n = 141, 42%; n = 117, 35% respectively) (Table 2).

**Table 2:** The use and favorability of the appointment systems*

System	n (%)	
	Use	Favorability
IVR	159 (47.89)	117 (35.24)
Web-based application	202 (60.84)	141 (42.47)
USSD	54 (16.26)	45 (13.56)
Other	21 (6.32)	29 (8.73)

*Since each participant could select more than one type of the systems, the sum of the percentages in “Use” column of the table is more than 100

**Fig. 1:**
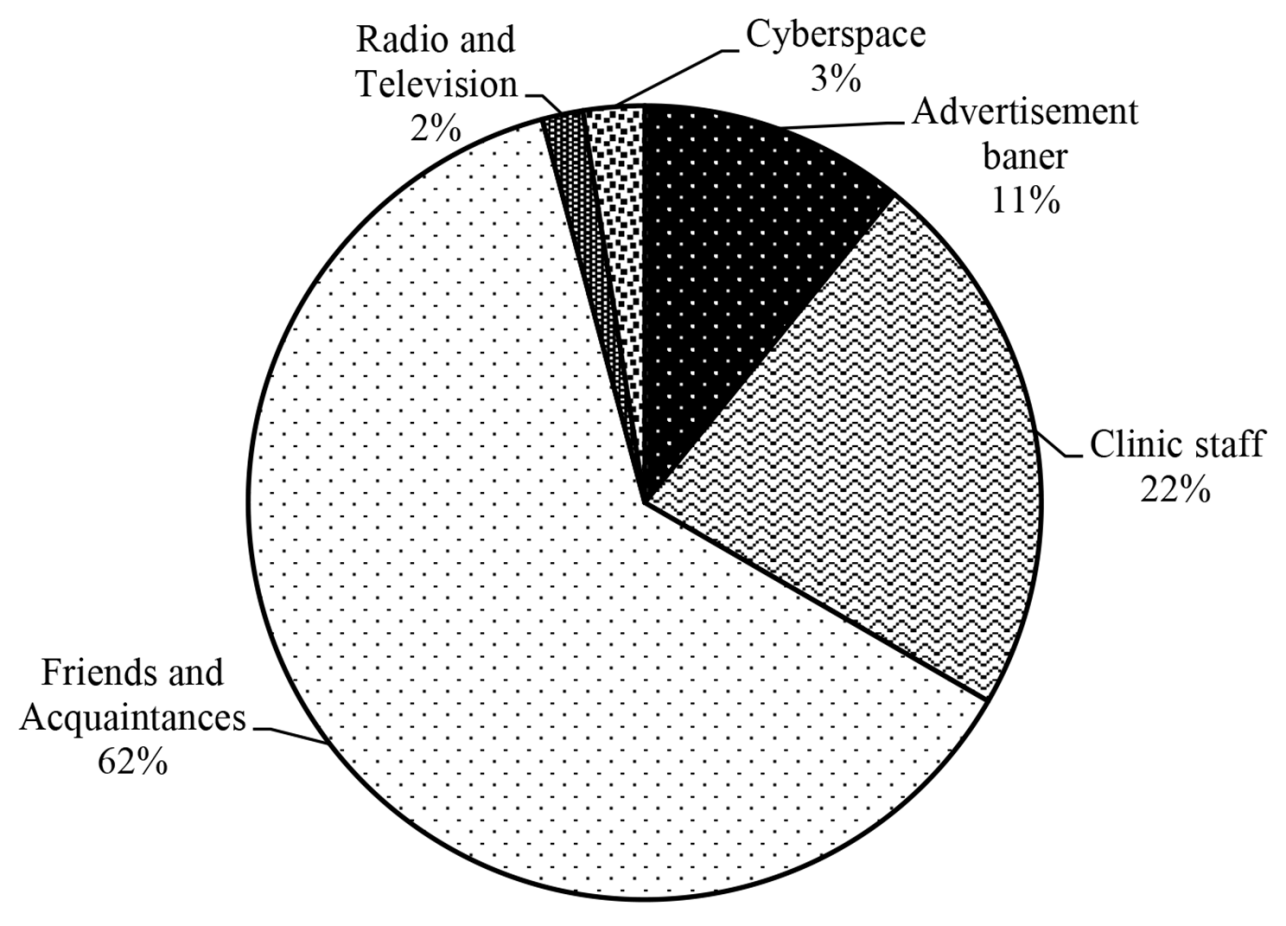
The ways the participants inform about the appointment systems

Thirty-six% (n = 120) of the participants announced that how they inform about the appointment systems. Among these participants, 62% (n = 75) stated that they get information from their friends and acquaintances. The least sources of the information were public media including radio and television (%2) (Fig. 1).

Forty-one percent (n = 137) of the participants had access to both the Internet and telephone tools (fixed and mobile) to arrange their appointments. Moreover, 34% (n = 113) of the participants had only access to the telephone and 21% (n = 70) had only access to the Internet. In general, 96% (n = 320) of participants had access to at least one Internet or telephone tools. There was a significant relationship between the system of interest and the tools available for arranging the appointment (p < 0.05, Pearson Chi-Square = 83.37). Among 113 participants who had access to the telephone, 56% (n = 63) chose IVR as favorable system among the others. Among 70 participants who had access to the Internet, 71% (n = 50) preferred web-based application compared to other systems. Moreover, among 137 participants who had access to both the Internet and telephone, 49% (n = 67) stated that they would rather arrange their appointment through the web-based application (Table 3).

**Table 3:** Cross-tabulation of interest regarding appointment system and access to required tools

Interest regarding appointment system	Access to required tools n (%)				
	Telephone	Internet	Both	None	Sum
IVR	63 (19)	11 (3.31)	39 (11.74)	4 (1.20)	117 (35.24)
Web-based application	22 (6.62)	50 (15.06)	67 (20.18)	2 (0.60)	141 (42.47)
USSD	18 (5.42)	4 (1.20)	23 (6.92)	0	45 (13.56)
Other	10 (3.01)	5 (1.50)	8 (2.40)	6 (1.80)	29 (8.73)
Sum	113 (34.03)	70 (21.08)	137 (41.26)	12 (3.61)	332 (100)

The participants’ overall satisfaction towards appointment systems, regardless of the type of system, was 49.12 ± 16.04 (out of 100). There was no significant relationship between the demographic characteristics of the participants and the satisfaction score (p > 0.05). The results of the ANOVA test showed that there was a significant relationship between the satisfaction when using the appointment systems and the type of system (p < 0.05). This means that the level of satisfaction with the USSD system when used was significantly higher than the other two systems (p = 0.03) (Table 4).

**Table 4:** The relationship between satisfaction of the participants and appointment systems

Appointment systems	satisfaction Mean ± SD					
	During the process	P	After making the appointment	P	Overall satisfaction	P
IVR	49.21 ± 16.37	0.03^*^	39.48 ± 39.33	0.62	47.47 ± 17.16	0.17
Web-based application	50.15±14.17		41.85 ± 37.48		48.65 ± 14.38	
USSD	58.67 ± 16.24		54.51 ± 42.93		57.91 ± 17.54	
All appointment systems (regardless of the type of system)	50.64 ± 15.41		42.21 ± 38.81		49.12 ± 16.04	

* Indicate p < 0.05, SD Standard Deviation

Seventy-one% (n = 236) of the participants preferred to receive a confirmation message after arranging the appointment. But only 52% (n = 174) of the participants stated that they had received a confirmation message. Of the participants, 332 individuals stated their viewpoints regarding the content of the confirmation message. They stated that the required information that should be included in the message is the date of the appointment (79%), Name and specialty of the provider (77%), the exact appointment time (73%), the exact address of the outpatient center (73%), and patient identity information (22%).

Regarding the best time for arranging the appointment via appointment systems, 52% (n = 171) of the participants preferred to have the possibility of 24 h responsive system. Of the participants, 15% (n = 50) chose the time interval from 6 am to 12 am, 18% (n = 61) from 12 am to 6 pm, 12% (n = 41) 6 pm to 12 pm, and 3% (n = 9) chose the time interval 12 pm to 6 am.

Forty-two% (n = 138) of the participants answered the question related to the problems concerning the appointment process and its system. The mentioned problems regarding appointment system include 37% (n = 51) failure of the system to save the appointment, 11% (n = 15) busy telephone line, 10% (n = 14) lack of insurance coverage due to miss-information, 9% (n = 13) mismatch between the arranged time and the actual time, 5% (n = 7) lack of existence of few specialists names in the list, 3% (n = 4) disconnection during arranging the appointment, 2% (n = 3) incorrect registration of details. The mentioned problems based on process include14% (n = 19) absence of the doctor on time, 5% (n = 7) limited time for making the appointment, 4% (n = 5) not accepting the appointment by admission personnel.

**Fig. 2:**
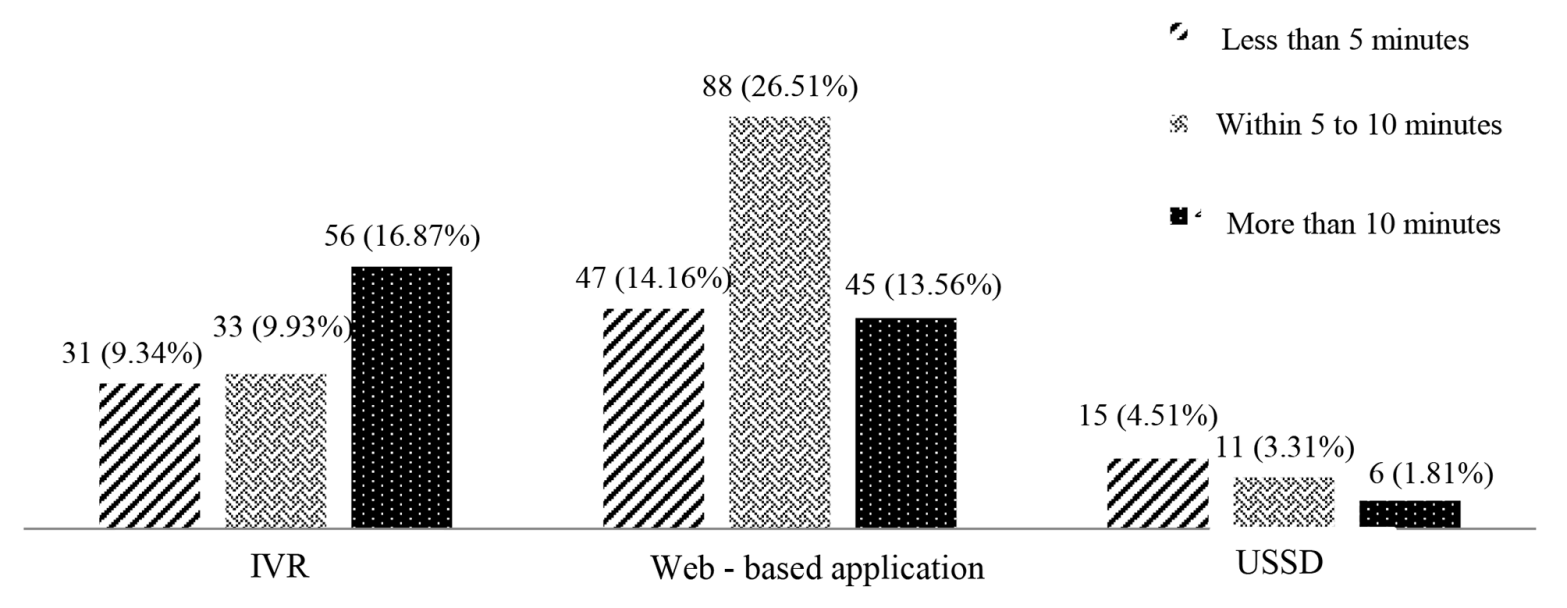
The average time for arranging the appointment

Figure 2 shows the average time that participants spent arranging their appointment. Approximately 40% (n = 132) of participants stated that their appointment process was successfully completed within 5 to 10 min, regardless of the type of appointment system.

## Discussion

### Principal findings

In this study, the overall satisfaction level of the participants with appointment systems, regardless of the type of system, was moderate. Among these systems, the participants had higher satisfaction with the USSD appointment system during the process of arranging the appointment. The web-based application and IVR systems were the most frequently used systems and were more favorable among the participants. More than half of the participants who had access to the telephone preferred the IVR appointment system, and most of those who had access to the Internet preferred the web-based application. Participants who had access to both of these tools preferred the web-based application. Most participants informed regarding the existence of these systems by friends and acquaintances.

Based on our findings, the satisfaction level of the participants with appointment systems is moderate. This result is in line with the findings of other studies, which have shown that the appointment system has a positive effect on patient’s satisfaction with receiving services, reducing no-shows, reducing staff workload, increasing waiting time [1, 8–11, 18] and getting the right time with the doctor and 24-hour access to the systems [19–21].

In this study, although participants preferred to use the web-based application, the satisfaction rate with the USSD system is higher. This result is in line with the results of the Barron, et al’s study [22], Users’ satisfaction with the USSD system was very high than the other appointment systems. The reasons for this higher satisfaction can be due to its accessibility (by simply dialing a number) and easy to use USSD, and it works on almost all cell phones [23, 24]. This system is sustainable and cost-effective support system to provide rural health care [25].

The results of this study indicated that the web-based application and IVR system are the most frequently used systems and more favorable among the participants. This result is consistent with the results of studies done by Yu, Heidari, Leung, Habibi, and Knight [9, 23, 24, 26, 27], which have shown that web-based application and telephone are the most popular appointment systems, and patients had satisfaction with these systems as they have benefits such as reducing the use of resource and staff, reducing waiting time and improving satisfaction.

In this study, more than half of the participants who had access to the telephone preferred the IVR appointment system, and most of those who had access to the Internet preferred the web-based application. This result is in line with the results of the study by Yu, et al’s [9] which showed that access to required tools and the understanding and ability to use appointment systems by the individuals are the strong motivators in using these systems. In the present study, the participants preferred to use the web-based application. Busy telephone lines and the need for several attempts to make appointments were the main problems of using the telephone to make an appointment. On the other hand, the use of the Internet-based appointment systems can not only be used as a tool to receive and provide health services, but also studies have shown that it can be used as a source of knowledge [28, 29]. Therefore, combining the Internet-based appointment systems with knowledge resources can increase the motivation of users to use these systems.

Based on our findings, most participants informed regarding the existence of these systems by friends and acquaintances. This result is in line with the finding of the study by Yu, et al’s [9] which showed that the suggestion of friends or family were the two main ways to inform about appointment systems. As being informed about the existence of appointment systems will increase the use of these systems and consequently will lead to satisfaction, it is necessary that after the implementation of these systems try to apply various methods to introduce them to the public.

Most of the participants in this study wanted to receive a confirmation message to ensure about the accuracy of the arranged appointment. However, due to financial constraints, sending text message after arranging the appointment is restricted to randomly selected patients as well all patients who arranged their appointment at specific time periods. Providing the appointment confirmation message and also the reminder message regarding the appointment time can lead to an increase in user satisfaction. This result is consistent with the findings of studies by Junod Perron and McClean [30, 31] which have shown that Short Message Service (SMS) reminders significantly increased satisfaction. In the study done by Nakhaee, et al’s [32], it has been stated that by sending a reminder SMS, it is possible to avoid office crowd due to forgetting the date or time of the appointment and reduce no-shows. Koshy, et al’s [33] also stated in their study that text messaging led to timely attendance and reduced patient waiting time.

The date of the appointment, the name of the care provider and her specialty, the exact time of the appointment, and the exact address of the center were determined as the most important content of the confirmation message by the participant in this study. Sending confirmation messages may increase patients’ assurance and providing this information will not only inform the patient about the correct process of making an appointment but will also reduce confusion and disruption on the day of the appointment [17, 19, 20, 34].

Based on our findings, appointment systems significantly reduce the time of arranging the appointment and saved the participants’ time. This result is consistent with the results of studies by Zhang, Cao, Knight, and Mohebbifar [21, 25, 27, 35], which have shown that appointment systems can effectively reduce waiting time.

The most frequent problems expressed by individuals are the failure of the system to save the appointment, the absence of the provider on time, constant busy telephone lines, the lack of insurance coverage, and the mismatch between the arranged appointment and the actual appointment. This result is in line with the results of study by Nakhaee, et al’s [32], which conducted a study to investigate the problems of appointment systems of physicians’ offices in Kerman from the perspective of secretaries and physicians. Busy telephone lines and lack of timely attendance of physicians in the office were among the problems reported in this study.

In this study, we had three limitations. The first one was the non-cooperation of people referring to the centers. Some people were not able to participate in the research due to lack of time, low literacy, and unfamiliarity with some appointment systems. Second, this research was conducted in several selected centers in Kerman; our findings may not be generalizable to other centers. Third, although mobile applications are one of the types of appointment systems, it was not implemented in this setting. So we could not evaluate mobile applications.

Future studies can increase the strength of evidence and the generalizability of the study by designing a study with a higher level of evidence (e.g., interventional studies). In future studies, the cost and time spent on these systems can also be compared together, and people’s satisfaction can be examined in different dimensions.

### Implications of the study

Surveying the use and satisfaction of users of appointment systems provides the possibility for health policymakers to review these systems to improve them and achieve maximum effectiveness. Also, by examining the causes of non-use and dissatisfaction with appointment systems and considering which systems are most used, these systems can be designed and implemented efficiently. While the web-based application and IVR are the most used and favorable systems among people, and they can significantly save patients time, but the level of satisfaction with such systems is not as expected. This may be due to the above mentioned problems. These problems can cause dissatisfaction among people. The results showed that despite the implementation of this system, participants were more informed regarding these systems through less formal sources. Therefore, while planning to solve problems to have more efficient systems; authorities should use the capacity of information resources such as medias and social networks to provide extensive information.

## Conclusion

The web-based application and IVR are the most frequently used systems and are morable among the participants. Most of the participants who have access to the telephone prefer the IVR appointment system, and most of those who have access to the Internet prefer the web-based application. Patients or those accompanying patients who have access to both of these tools prefer the web-based application. But despite the availability of the necessary infrastructure for patients or those accompanying patients to use appointment system, the overall satisfaction level of the participants with appointment systems, regardless of the type of system, is moderate.

Therefore, evaluating appointment systems and solving their problems to have more efficient systems can be effective in increasing people’s satisfaction. Given that most participants were informed of the existence of these systems by friends and acquaintances, it is suggested that officials use the maximum capacity of information resources such as media, social network and health staff to provide extensive information.

## Data Availability

The data generated and analyzed during this study are available from the corresponding author on reasonable request.

## List of abbreviations

IVR: Interactive Voice Response
USSD: Unstructured Supplementary Service Data
KUMS: Kerman University of Medical Sciences
ANOVA: Analysis of Variance
SMS: Short Message Service
SD: Standard Deviation
DTMF: Dual Tone Multi Frequency
GSM: Global System for Mobile Communications
